# Examining the longitudinal influence of loneliness on healthcare utilization: evidence from Taiwan’s national health insurance data

**DOI:** 10.1186/s12877-025-06088-0

**Published:** 2025-06-05

**Authors:** Shiau-Fang Chao, Hui-Chuan Hsu, Chia-Le Yen, Bo-Yu Chen

**Affiliations:** 1 Department of Social Work, National Taiwan University, No 1, Section 4, Roosevelt Road, Daan District, Taipei, 10617 Taiwan; 2 School of Public Health, Taipei Medical University, New Taipei City, Taiwan; 3 Department of Economics, National Taiwan University, Taipei, Taiwan; 4 Vancouver School of Economics, University of British Columbia, Vancouver, Canada

**Keywords:** Emotional loneliness, Social loneliness, Health service utilization, Longitudinal, Taiwan

## Abstract

**Background:**

This study combines a nationally representative sample from Taiwan with four years of National Health Insurance (NHI) data to explore the distinctive impact of emotional and social loneliness on health service utilization, including outpatient visits for mental health, general outpatient visits, emergency room (ER) visits, and hospitalization.

**Methods:**

Data were drawn from the 2015 Taiwan Longitudinal Survey on Aging (TLSA) and merged with participants’ NHI records from 2015 to 2018. The analysis used logistic regression for binary outcomes and negative binomial regression for counts.

**Results:**

Results show that higher emotional loneliness in 2015 was associated with increased outpatient mental health visits over time and more general outpatient visits within the same year. Conversely, higher social loneliness in 2015 reduced the likelihood of seeking ER care in 2015.

**Conclusions:**

By merging national data and distinguishing emotional from social loneliness, this study offers insights into their differential impacts on healthcare utilization among older adults in Taiwan. It emphasizes the importance of addressing loneliness to improve physical and mental well-being and optimize the effective utilization of healthcare resources.

## Background

The prevalence of loneliness in the older population shows varies depending on regions, measurement methods, and sample characteristics, ranging from below 6% to over 40% [1]. As a whole, the older population generally experiences higher levels of loneliness compared to younger and middle-aged individuals, regardless countries and areas [1]. Loneliness in later life has become a significant public concern due to its negative impact on health outcomes, including higher mortality rates, high blood pressure, increased cholesterol levels, coronary disease, and declined functional or cognitive functioning [2, 3]. Loneliness is also associated with diminished behavioral, mental, social health, and quality of life [1].

Existing studies have primarily focused on the health impacts of loneliness, with less attention given to examining how loneliness influences different types of healthcare utilization [4]. Additionally, a limited number of existing studies reveal conflicting outcomes. Tang et al. (2022) conducted a review article examining seven studies from 2000 to 2021 that identified individual determinants of emergency room (ER) utilization in older adults [5]. Drawing on findings from Wee et al. (2019), Tang et al. (2022) used meta-analytic methods to demonstrate that loneliness was associated with higher rates of ER visits among older adults residing in public rental housing in Singapore [5]– [6].

As for outpatient visits, the research findings on the relationship with loneliness are inconsistent. Some studies using samples from China, the US, and Canada reported a positive association between loneliness or higher levels of loneliness and increased outpatient visit frequency [7–10], while others conducted in Singapore or the US found a negative correlation between loneliness and healthcare utilization [3, 11].

The results of current studies on hospitalization also vary across different findings. Gerst-Emerson and Jayawardhana (2015) defined chronic loneliness as being lonely in both the 2008 and 2012 h data in the US and found that loneliness reported in 2008 was significantly related to hospitalization in 2012. However, chronic loneliness was not significantly associated with hospitalization in studies from China or other US studies [7]– [8, 10]. In contrast, Shaw et al. (2017) indicate that in a US sample, loneliness could hinder access to inpatient care, leading to lower Medicare inpatient reimbursement for lonely beneficiaries compared to non-lonely beneficiaries with similar health statuses. Reimbursement calculated by dividing the total Medicare inpatient reimbursement by the number of months of follow-up claims data for each individual [11]. Using a sample from Canada, Newall et al. (2014) considered loneliness as a predictor of both hospitalization (0/1) and rehospitalization over a 2.5-year period. They found that loneliness was not associated with the odds of hospitalization; however, being lonely was linked to higher odds of being rehospitalized two or more times. This suggests that loneliness may negatively impact recovery from illness or surgery, increasing the likelihood of multiple hospitalizations [9]. The findings from Shaw et al. (2017) and Newall et al. (2014) demonstrate that hospitalization utilization can be measured in various ways—such as average inpatient reimbursement, hospitalization frequency, or binary admission status—each potentially yielding different outcomes [9, 11]. The differing results on healthcare use outcomes in the studies discussed above emphasize the need for further research to enhance our comprehensive understanding of how loneliness impacts healthcare outcomes.

Upon reviewing the available literature on loneliness and healthcare utilization, several limitations that could contribute to discrepancies in current findings should be addressed. First, the measurement of loneliness varies among studies, with the 3-item UCLA loneliness scale being the most commonly used [5, 11]– [12]. The UCLA loneliness scale assesses an individual’s subjective evaluation of feeling excluded, isolated, and lacking companionship [13], primarily reflecting the social aspect of loneliness. Additionally, a few prior studies adopted a single-item measurement approach and categorized loneliness as a binary response [7, 9]– [10].

However, according to Weiss (1973), loneliness consists of two distinct components: emotional and social loneliness. Emotional loneliness is characterized by intense feelings of emptiness, abandonment, and forlornness due to the absence of intimate relationships. Social loneliness refers to the subjective evaluation of deficiencies in the broader social network of relationships, and these two aspects should be distinguished from each other [14]– [15]. To our knowledge, no study has comprehensively incorporated these two components of loneliness to investigate their influences on healthcare utilization. Second, the majority of studies investigating healthcare utilization rely on subjective self-report data [3, 8], which may be influenced by recall bias. In other words, except for a few exceptional studies [9, 11, 16], the utilization of objective sources such as medical records or administrative data is limited. Moreover, there is a wide variation in the measurement intervals, spanning from a few weeks to several years without a consistent timeframe. Third, based on our understanding, the existing literature on the relationship between loneliness and healthcare services primarily focuses on outcomes such as inpatient admissions, outpatient visits, ER visits, or re-hospitalization. The influence of loneliness on mental health service utilization has been understudied. Fourth, with few exceptions [3, 8, 11, 17], the existing literature predominantly consists of cross-sectional studies, making it difficult to ascertain the short-term and long-term effects of loneliness on different types of healthcare services. Fifth, most of the current literature is derived from Western samples, and the differences in the relationship between loneliness and healthcare utilization may extend beyond individual behavior to variations within different cultural and social contexts [3]. Therefore, further research is needed to expand this topic to non-Western samples.

As of the end of 2022, individuals aged 65 and above was approximately 4 million, accounting for 17.56% of Taiwan’s total population, and this proportion is expected to grow rapidly, reaching 20.8% by 2026 [18]. According to the 2017 Senior Citizen Condition Survey, 17.3% of individuals aged 65 and above in Taiwan sometimes feel lonely, while 3.4% feel lonely frequently [19]. Su et al. (2023) also reported that older adults in Taiwan had a mean loneliness score of 3.86 (1.74 for emotional loneliness, 2.13 for social loneliness), which is notably higher than scores from European, American, Japanese, and Singaporean samples [20]. These rates are slightly higher than those in China and comparable to levels in Eastern and Southern Europe [1, 7], possibly due to cultural differences in relationship expectations, as older adults in Taiwan may anticipate more filial piety from their children, such as co-residence or constant companionship, leading to loneliness when these expectations are unmet [20]. Meanwhile, Taiwan’s National Health Insurance (NHI), implemented in 1995, is a comprehensive and universal healthcare system that ensures coverage, promotes affordability, and facilitates accessibility to medical services for the entire population of the country [21]. In 2020, individuals aged 65 and above utilized 39.8% of the total healthcare expenditures in Taiwan’s NHI system. As the aging trend continues, this proportion is expected to increase gradually [22]. Given the high prevalence of loneliness among older adults in Taiwan, along with the accessibility and affordability of healthcare services, the country provides a unique setting to explore the link between loneliness and healthcare utilization. Elevated levels of loneliness may contribute to increased healthcare use within the NHI system, potentially due to its negative impacts on mental and physical health or the risk of excessive medical use. Therefore, further research is needed to clarify these associations.

This study seeks to answer the following research questions: (1) How do the emotional and social components of loneliness affect various forms of healthcare utilization in Taiwan? (2) What are the short- and long-term impacts of these two components of loneliness on healthcare services? This study advances current literature by combining a nationally representative sample in Taiwan with a four-year NHI records to examine the influence of loneliness on health service utilization in both the short and long term, including outpatient visits for mental health issues, the frequency of general outpatient and ER visits, and the likelihood of ER visits or hospitalization. Expanding on prior studies, this study differentiates loneliness into emotional and social components to explore their distinct effects on the aforementioned various types of healthcare utilization. Given the complexity of calculating healthcare expenditures across countries, this study focuses on whether services are used and the frequency of service utilization, rather than costs, to enhance the comparability of its findings with other studies. Moreover, the accessibility and affordability of Taiwan’s NHI system provide a unique opportunity to examine whether loneliness is a risk factor for excessive medical utilization, compared to countries with less accessible or more burdensome healthcare resources.

## Methods

### Data and sample

The data utilized in this study were obtained from the Taiwan Longitudinal Survey on Aging (TLSA) combined with the NHI data. TLSA is a nationally representative sample of older individuals in Taiwan. The TLSA began in 1989 with participants aged 60 years or older, taken through probability sampling. Follow-up surveys were conducted every 3 to 4 years, with additional cases included in subsequent waves. To address issues of sample aging and attrition, the TLSA added on samples of individuals aged 50 and above in 1996, 2003, and 2015, respectively, according to the original sampling methodology. The questionnaires were administered through face-to-face interviews. Due to regulations from Taiwan’s Ministry of Health and Welfare, NHI data can only be linked with TLSA data from the same year and the following three years. Additionally, only one wave of TLSA data can be used for NHI linkage to avoid the risk of identifying individuals through longitudinal and cross-database linkages. Therefore, the sample utilized in this study was drawn from the 2015 survey, as the variable of interest, loneliness, was evaluated exclusively in the 2015 wave.

Participants who provided consent to connect their personal TLSA data with NHI data, responded to the questionnaire without the use of a proxy, and had no missing data on the variables utilized in this study were included for analysis. A total of 500 participants were excluded due to death between 2015 and 2018, use of a proxy, or missing data. The final sample size was 6,472, and their 2015 TLSA data were merged with NHI data from 2015 to 2018. Further analyses showed that those excluded from the analyses were older, predominantly male, with higher social and emotional loneliness, greater ADL difficulty, and more diseases. In 2015, they also had more outpatient visits for mental illness or general reasons, as well as higher rates of emergency visits and hospitalizations.

### Measures

#### Dependent variables: healthcare utilization

The dependent variables of this study encompassed four NHI utilization records, representing: (1) outpatient visits for mental illness; (2) number of outpatient visits for general reasons; (3) ER visits; (4) hospital admission.

Outpatient visits for mental illness was accessed by whether the participant sought outpatient service in the given year due to mental and behavioral disorders listed in the International Classification of Diseases, 10th Revision (ICD-10) under the codes F00-F99 (*yes = 1*,* no = 0*) [23]. Number of outpatient visits was measured by the total number of outpatient visits made by respondents in the given year, regardless of the medical specialty, but excluding those made for mental illness reasons. ER visits were measured based on whether an individual visited the ER during the same calendar year. Hospital admission was measured using whether individuals were hospitalized for any reason during the given year (*yes = 1*,* no = 0*).

#### Independent variables: loneliness

The six-item De Jong-Gierveld Loneliness Scale [24] was employed to assess loneliness. Emotional loneliness was measured with three items (“I experience a sense of emptiness around me,” " I miss having people around me,” and " often, I feel rejected”), while social loneliness was assessed using the other three items (“There are plenty of people that I can dependent on if I’m in trouble,” " There are many people that I can count on completely,” and " There are enough people that I feel close to”). Participants indicated their responses on a scale of *no* (score 0), *more or less* (score 1), and *yes* (score 2). The three items in social loneliness were reversely recoded. The total score for emotional loneliness ranged from 0 to 6, while the total score for social loneliness also ranged from 0 to 6, with higher scores indicating greater levels of emotional or social loneliness.

#### Other covariates

The selection of covariates in this study was based on the Andersen model [25], which includes predisposing factors, enabling factors, and need factors. The Andersen model is suitable for selecting covariates in this study, as it offers a comprehensive framework to account for the multifactorial influences on healthcare utilization, helping to control for potential confounders and ensuring a more accurate analysis of the relationship between loneliness and healthcare usage [25]. Predisposing factors refer to basic demographic variables and tendency to use healthcare of individuals, such as age, gender (*0 = female; 1 = male*), and education (elementary, junior high school, senior high school, or college and above). Enabling factors encompass to economic, family, or community resources that may influence individuals’ access to healthcare services, including residential area (large city, big city, small city, township, or country), marital status (*0 = with no spouse; 1 = with a spouse*), living arrangement (*0 = not living alone; 1 = living alone*), and financial sufficiency, scored from 1 (*very insufficient*) to 4 (*very sufficient*). Need factors refer to physical and mental health needs that can affect healthcare utilization, including cognitive impairment, ADL difficulty, and the number of diseases. With SPMSQ Error was assessed utilizing the 10-item Short Portable Mental Status Questionnaire (SPMSQ). Example questions asked for participants’ address, interview date and location, day of the week, age, mother’s maiden name, names of the current and previous presidents, birth date, and counting backward from 20 by threes [26]. All questions answered correctly coded as 0 and one or more questions answered incorrectly coded as 1. ADL difficulty was measured by summing the difficulty levels of performing six ADL tasks, including bathing, dressing, eating, getting up, indoor walking, and using the toilet [27]. Each task was assigned a score ranging from 0 (*no difficulty*) to 3 (*unable to perform*), with a higher score indicating greater ADL difficulty. The number of diseases represented self-reported medical conditions diagnosed by physicians for 20 commonly observed diseases in older population, such as hypertension, diabetes, heart disease, cancer, chronic bronchitis, emphysema, asthma, arthritis, and more. The cumulative count of these chronic diseases was utilized.

### Statistical analysis

Sample descriptive statistics were first computed, followed by examining the correlation between emotional and social loneliness in 2015 and the four dependent variables from 2015 to 2018 (results not shown). When the dependent variables were outpatient visits for mental illness, ER visits or hospital admission, the analytic procedure involved utilizing multivariable logistic regression, considering the binary nature of these three variables. Negative binomial regression was used for modeling the count outcome variable (i.e., number of outpatient and ER visits). The independent variables included emotional and social loneliness, along with other predisposing, enabling, and need factors in 2015. For the various regression modeling mentioned above, separate analyses were conducted using the independent variables and control variables in 2015, and the dependent variables from 2015 to 2018. The primary objective of this study was to examine the causal relationship between loneliness and healthcare utilization, rather than to generate descriptive population estimates. Following Solon et al. (2015), when key confounding variables are properly controlled, unweighted regression analyses are generally preferable because they minimize potential selection bias and produce more efficient estimates [28]. In this study, covariate selection was guided by the Andersen model, which comprehensively accounts for predisposing, enabling, and need factors, helping to control for potential confounders. Thus, this study did not apply sample weights in the analyses. Lastly, all analyses were conducted using R (version 4.1.3).

## Results

Table 1 presents the characteristics of the sample in 2015, as well as their healthcare utilization from 2015 to 2018. The rate of SPMSQ errors was 25.3%, with most participants answering 1–2 items incorrectly. Approximately 1% met the criteria for moderate cognitive impairment (5–7 incorrect answers), and 1.6% met the criteria for severe cognitive impairment (8 or more incorrect answers). As noted earlier, respondents requiring proxies due to cognitive inability were excluded from this study. The mean emotional and social loneliness scores in 2015 were 1.71 (SD = 1.06) and 1.96 (SD = 1.70), respectively. As for healthcare utilization, in 2015, approximately 12.3% of the sample had visited outpatient due to mental illness. From 2016 to 2018, this proportion remained relatively stable at around 12 to 13%. From 2015 to 2018, the number of outpatient visits consistently remained in the range of 25 to 27 times each year. Regarding the proportion of participants with ER visit records in the same year, the percentage was lower in 2015 at 19.3%, but from 2016 onwards, it consistently ranged between 20% and 22%. The prevalence of hospital admissions increased from 12.5% in 2015 to 14.7% in 2018.

**Table 1:** Means, standard deviations, characteristics of the sample (*N* = 6,472)

Variables	Mean (SD) or %
**Independent Variables in 2015**	
**Emotional loneliness** (0–6)	1.71 (1.06)
**Social loneliness** (0–6)	1.96 (1.70)
**Age** (50–100)	65.89 (9.78)
**Gender**	
-Female	51.9%
-Male	48.1%
**Education**	
-Elementary school or below	50%
-Junior high school	15.9%
-Senior high school	24.9%
-College and above	9.2%
**Residential area**	
- Large city	59.9%
- Big city	7.3%
- Small city	10.5%
- Township	7.8%
- Country	14.5%
**Marital status**	
-With a spouse	73%
**Living alone** (Yes)	8.5%
**Financial status**	
-Very insufficient	3.2%
-Insufficient	14.2%
-Sufficient	71.2%
- Very sufficient	11.4%
**With SPMSQ Error** (Yes)	25.3%
**ADL difficulty** (0–18)	0.27 (1.68)
**Number of disease** (0–16)	1.21 (3.82)
**Dependent variables**	2 (1.80)
Outpatient visits for mental illness (Yes)	
-2015	12.3%
-2016	12.0%
-2017	12.7%
-2018	13.2%
Number of outpatient visits for general reasons	
-2015 (0-155)	25.71 (20.84)
-2016 (0-150)	26.53 (21.12)
-2017 (0-138)	26.76 (21.06)
-2018 (0-133)	27.28 (20.91)
ER visits (Yes)	
-2015	19.3%
-2016	21.0%
-2017	20.9%
-2018	22.6%
Hospital admission (Yes)	
-2015	12.5%
-2016	12.2%
-2017	13.5%
-2018	14.7%

Note: SPMSQ = Short Portable Mental Status Questionnaire; ADL = Activities of daily living; ER = Emergency Room

Table 2 presents the results of the multivariable logistic regression, with outpatient visits for mental illness as the outcome. Participants with higher levels of both emotional and social loneliness in 2015 consistently showed a greater likelihood of seeking outpatient visits for mental illness during the same year (OR: emotional loneliness = 1.081, *p* <.05; social loneliness = 1.094, *p* <.05). Only higher levels of emotional loneliness in 2015 had a significant longitudinal impact on outpatient visits for mental illness in the years 2016 (OR = 1.123, *p* <.01), 2017 (OR = 1.119, *p* <.01), and 2018 (OR = 1.087, *p* <.05), while social loneliness did not show a similar effect.

**Table 2:** Multivariable logistic analyses for loneliness and outpatient visits for mental illness

Independent Variables in 2015	2015	2016	2017	2018
	OR (95% CI)	OR (95% CI)	OR (95% CI)	OR (95% CI)
**Emotional loneliness**	1.081 (1.003, 1.165)*	1.123 (1.041, 1.212)**	1.119 (1.039, 1.206)**	1.087 (1.009, 1.170)*
**Social loneliness**	1.094 (1.011, 1.184)*	1.045 (0.964, 1.133)	1.047 (0.967, 1.133)	1.023 (0.946, 1.106)
**Age**	0.984 (0.897, 1.080)	1.102 (1.005, 1.209)*	1.194 (1.091, 1.306)***	1.198 (1.097, 1.308)***
**Gender** (ref. Female)	0.843 (0.778, 0.914)***	0.866 (0.798, 0.939)***	0.861 (0.795, 0.932)***	0.862 (0.797, 0.932)***
**Education** (ref. Elementary)				
-Junior high school	1.114 (1.025, 1.209)*	1.086 (0.998, 1.180)	1.118 (1.029, 1.212)**	1.114 (1.028, 1.206)**
-Senior high school	1.120 (1.024, 1.224)*	1.099 (1.005, 1.201)*	1.179 (1.082, 1.285)***	1.131 (1.038, 1.231)**
-College and above	1.103 (1.012, 1.200)*	1.067 (0.978, 1.162)	1.014 (0.925, 1.106)	1.026 (0.940, 1.117)
**Residential area** (ref. Large city**)**				
- Big city	0.998 (0.923, 1.074)	1.054 (0.979, 1.131)	1.046 (0.973, 1.121)	1.023 (0.950, 1.097)
- Small city	1.002 (0.926, 1.081)	0.962 (0.885, 1.041)	0.960 (0.879, 1.029)	0.985 (0.911, 1.061)
- Township	0.975 (0.897, 1.055)	0.945 (0.865, 1.025)	0.942 (0.886, 1.036)	0.999 (0.925, 1.076)
- Country	1.007 (0.929, 1.089)	1.000 (0.922, 1.083)	0.996 (0.919, 1.076)	1.036 (0.959, 1.116)
**Marital status** (ref. No spouse)	1.025 (0.942, 1.118)	1.060 (0.972, 1.159)	1.068 (0.981, 1.165)	1.039 (0.956, 1.130)
**Living alone** (ref. No)	1.058 (0.978, 1.142)	1.043 (0.962, 1.128)	1.071 (0.991, 1.155)	1.069 (0.991, 1.150)
**Financial status** (ref. Very insufficient**)**				
-Insufficient	0.936 (0.816, 1.080)	0.920 (0.798, 1.068)	0.952 (0.826, 1.105)	1.050 (0.904, 1.232)
-Sufficient	0.852 (0.721, 1.016)	0.878 (0.739, 1.054)	0.899 (0.757, 1.079)	1.034 (0.861, 1.259)
- Very sufficient	0.936 (0.817, 1.076)	0.934 (0.812, 1.080)	0.990 (0.862, 1.142)	1.103 (0.955, 1.282)
**With SPMSQ Error** (ref. No)	0.887 (0.818, 0.963)**	0.970 (0.892, 1.055)	0.972 (0.896, 1.056)	0.926 (0.856, 1.003)
**ADL difficulty**	0.979 (0.898, 1.059)	0.986 (0.905, 1.067)	1.039 (0.961, 1.116)	1.015 (0.937, 1.092)
**Number of disease**	1.333 (1.235, 1.439)***	1.299 (1.202, 1.403)***	1.207 (1.117, 1.302)***	1.206 (1.118, 1.299)***
**Observations**	6,472	6,472	6,472	6,472
Log Likelihood	-2,349.554	-2,316.364	-2,400.685	-2,476.630
AIC	4,739.107	4,672.727	4,841.370	4,993.260

Note: OR = Odds Ratio; CI = Confidence Interval; SPMSQ = Short Portable Mental Status Questionnaire; ADL = Activities of daily living; AIC = Akaike Information Criterion

***p <.001.** *p*<.01. * *p*<.05

Table 3 presents the results of negative binomial regression, with the number of outpatient visits for general reasons as the dependent variable. The higher levels of emotional loneliness in 2015 were positively associated with increased occurrence of outpatient visits in 2015 (OR = 1.021, *p* <.05), even after considering other predisposing, enabling, and need factors. However, social loneliness showed no significant correlation with the number of outpatient.

**Table 3:** Negative binomial regression analyses for loneliness and number of outpatient visits for general reasons

Independent Variables in 2015	2015	2016	2017	2018
	OR (95% CI)	OR (95% CI)	OR (95% CI)	OR (95% CI)
**Emotional loneliness**	1.021 (1.001, 1.041) *	1.019 (0.999, 1.039)	1.007 (0.988, 1.026)	1.011 (0.991, 1.032)
**Social loneliness**	1.001 (0.987, 1.015)	0.997 (0.973, 1.021)	0.992 (0.971, 1.013)	0.985 (0.966, 1.005)
**Age**	1.152 (1.125, 1.180)***	1.136 (1.109, 1.163)***	1.141 (1.114, 1.168)***	1.117 (1.091, 1.143)***
**Gender** (ref. Female)	0.929 (0.910, 0.948)***	0.937 (0.918, 0.956)***	0.939 (0.920, 0.958)***	0.943 (0.925, 0.962)***
**Education** (ref. Elementary)				
-Junior high school	0.991 (0.970, 1.012)	0.989 (0.969, 1.009)	0.989 (0.969, 1.010)	0.989 (0.969, 1.010)
-Senior high school	0.990 (0.968, 1.012)	0.987 (0.964, 1.010)	0.981 (0.959, 1.004)	0.981 (0.959, 1.003)
-College and above	0.982 (0.961, 1.003)	0.982 (0.961, 1.003)	0.981 (0.961, 1.002)	0.970 (0.950, 0.991)**
**Residential area** (ref. Large city**)**				
- Big city	1.000 (1.000, 1.000)	1.004 (0.984, 1.025)	1.002 (0.984, 1.020)	0.992 (0.974, 1.011)
- Small city	0.988 (0.969, 1.007)	0.983 (0.964, 1.002)	0.982 (0.963, 1.002)	0.989 (0.971, 1.008)
- Township	1.019 (0.999, 1.040)	1.008 (0.989, 1.027)	1.009 (0.989, 1.029)	1.008 (0.989, 1.027)
- Country	1.014 (0.994, 1.035)	1.015 (0.994, 1.036)	1.011 (0.991, 1.032)	1.016 (0.996, 1.037)
**Marital status** (ref. No spouse)	1.036 (1.013, 1.059)**	1.039(1.016, 1.062) ***	1.057 (1.034, 1.081)***	1.045 (1.023, 1.068)***
**Living alone** (ref. No)	0.994 (0.972, 1.017)	0.987 (0.967, 1.007)	0.995 (0.974, 1.017)	0.997 (0.976, 1.018)
**Financial status** (ref. Very insufficient**)**				
-Insufficient	1.025 (0.983, 1.069)	1.017 (0.975, 1.061)	1.015 (0.974, 1.058)	1.029 (0.988, 1.072)
-Sufficient	1.002 (0.947, 1.060)	0.997 (0.950, 1.046)	0.984 (0.935, 1.035)	1.018 (0.969, 1.070)
- Very sufficient	0.999 (0.946, 1.055)	0.996 (0.955, 1.038)	0.985 (0.946, 1.026)	1.016 (0.976, 1.057)
**With SPMSQ Error** (ref. No)	1.028 (1.006, 1.050)*	1.028 (1.006, 1.051)*	1.034 (1.012, 1.057)**	1.040 (1.018, 1.062)***
**ADL difficulty**	1.027 (1.004, 1.051)*	1.016 (0.993, 1.039)	1.011 (0.989, 1.034)	1.012 (0.989, 1.035)
**Number of disease**	1.324 (1.296, 1.352)***	1.301 (1.274, 1.329)***	1.276 (1.249, 1.303)***	1.251 (1.225, 1.277)***
Observations	6,472	6,472	6,472	6,472
Log Likelihood	-26815.498	-27085.999	-27150.835	-27273.397
AIC	53672.996	54213.998	54343.671	54588.793

Note: OR = Odds Ratio; CI = Confidence Interval; SPMSQ = Short Portable Mental Status Questionnaire; ADL = Activities of daily living; ER = Emergency Room; AIC = Akaike Information Criterion. ***p <.001. ** *p*<.01. * *p*<.05

Differing from the previous analysis of outpatient visits, Table 4 reveals the results of a multivariable logistic regression analysis, indicating that an increase in social loneliness in 2015 was associated with a significant decrease in the odds of ER visits at the same year (OR = 0.930, *p* <.05).

**Table 4:** Multivariable logistic analyses for loneliness and ER visits

Independent Variables in 2015	2015	2016	2017	2018
	OR (95% CI)	OR (95% CI)	OR (95% CI)	OR (95% CI)
**Emotional loneliness**	1.008 (0.945, 1.075)	1.022 (0.960, 1.087)	1.010 (0.949, 1.076)	0.989 (0.930, 1.051)
**Social loneliness**	0.930 (0.869, 0.995)*	0.968 (0.907, 1.034)	1.043 (0.977, 1.114)	0.974 (0.913, 1.038)
**Age**	1.109 (1.027, 1.197)**	1.159 (1.076, 1.248)***	1.211 (1.124, 1.305)***	1.229 (1.144, 1.321)***
**Gender** (ref. Female)	1.042 (0.975, 1.115)	1.024 (0.959, 1.092)	0.982 (0.919, 1.048)	1.053 (0.988, 1.122)
**Education** (ref. Elementary)				
-Junior high school	0.963 (0.897, 1.032)	0.957 (0.893, 1.023)	0.981 (0.916, 1.050)	0.971 (0.909, 1.037)
-Senior high school	0.907 (0.841, 0.978)*	0.888 (0.825, 0.956)**	0.928 (0.862, 0.999)*	0.942 (0.878, 1.011)
-College and above	0.889 (0.820, 0.960)**	0.914 (0.849, 0.983)*	0.902 (0.834, 0.972)**	0.841 (0.777, 0.908)***
**Residential area** (ref. Large city**)**				
- Big city	1.028 (0.962, 1.095)	1.031 (0.968, 1.096)	1.023 (0.959, 1.090)	1.014 (0.953, 1.078)
- Small city	1.089 (1.022, 1.158)**	1.021 (0.958, 1.087)	1.074 (1.008, 1.142)*	1.027 (0.965, 1.092)
- Township	1.092 (1.027, 1.160)**	1.100 (1.037, 1.165)**	1.115 (1.050, 1.182)***	1.077 (1.015, 1.141)*
- Country	1.067 (1.000, 1.138)*	1.031 (0.968, 1.098)	1.139 (1.071, 1.212)***	1.064 (1.001, 1.131)*
**Marital status** (ref. No spouse)	0.988 (0.920, 1.061)	0.975 (0.910, 1.045)	1.026 (0.957, 1.101)	0.927 (0.867, 0.991)*
**Living alone** (ref. No)	1.020 (0.954, 1.090)	0.991 (0.928, 1.057)	1.034 (0.968, 1.102)	0.991 (0.930, 1.055)
**Financial status** (ref. Very insufficient**)**				
-Insufficient	1.064 (0.935, 1.215)	0.979 (0.866, 1.110)	1.069 (0.944, 1.217)	0.981 (0.868, 1.111)
-Sufficient	0.992 (0.848, 1.171)	0.927 (0.798, 1.082)	1.007 (0.864, 1.181)	0.980 (0.845, 1.142)
- Very sufficient	0.931 (0.819, 1.063)	0.941 (0.834, 1.064)	0.984 (0.870, 1.117)	0.928 (0.823, 1.049)
**With SPMSQ Error** (ref. No)	0.927 (0.866, 0.992)*	0.940 (0.880, 1.004)	0.952 (0.891, 1.018)	0.938 (0.880, 1.001)
**ADL difficulty**	1.093 (1.025, 1.165)**	1.092 (1.025, 1.162)**	1.111 (1.043, 1.183)**	1.063 (0.998, 1.132)
**Number of disease**	1.323 (1.239, 1.412)***	1.237 (1.160, 1.318)***	1.290 (1.210, 1.375)***	1.267 (1.190, 1.349)***
Observations	6,472	6,472	6,472	6,472
Log Likelihood	-3,064.081	-3,226.518	-3,178.037	-3,327.591
AIC	6,168.162	6,493.037	6,396.074	6,695.182

Note: OR = Odds Ratio; CI = Confidence Interval; SPMSQ = Short Portable Mental Status Questionnaire; ADL = Activities of daily living; ER = Emergency Room; AIC = Akaike Information Criterion. ***p <.001. ** *p*<.01. * *p*<.05

Unlike the analysis results presented in Tables 2, 3 and 4, the multivariable logistic regression results in Table 5 showed that neither emotional nor social loneliness was significantly associated with hospital admission in any of the years studied.

**Table 5:** Multivariable logistic analyses for loneliness and hospital admission

Independent Variables in 2015	2015	2016	2017	2018
	OR (95% CI)	OR (95% CI)	OR (95% CI)	OR (95% CI)
**Emotional loneliness**	1.007 (0.933, 1.087)	0.983 (0.910, 1.062)	1.008 (0.936, 1.086)	1.033 (0.962, 1.110)
**Social loneliness**	0.932 (0.858, 1.011)	0.978 (0.901, 1.061)	1.003 (0.927, 1.085)	0.939 (0.870, 1.013)
**Age**	1.123 (1.026, 1.230)*	1.224 (1.117, 1.339)***	1.217 (1.114, 1.328)***	1.266 (1.164, 1.376)***
**Gender** (ref. Female)	1.155 (1.065, 1.253)***	1.137 (1.048, 1.234)**	1.064 (0.984, 1.151)	1.172 (1.087, 1.264)***
**Education** (ref. Elementary)				
-Junior high school	0.899 (0.823, 0.981)*	0.971 (0.891, 1.055)	0.926 (0.850, 1.005)	0.935 (0.862, 1.011)
-Senior high school	0.888 (0.810, 0.972)*	0.871 (0.793, 0.955)**	0.903 (0.826, 0.985)*	0.890 (0.818, 0.969)**
-College and above	0.888 (0.807, 0.973)*	0.841 (0.759, 0.926)***	0.841 (0.761, 0.925)***	0.812 (0.736, 0.892)***
**Residential area** (ref. Large city**)**				
- Big city	1.033 (0.955, 1.114)	0.987 (0.908, 1.068)	1.016 (0.939, 1.095)	1.000 (0.927, 1.075)
- Small city	0.977 (0.898, 1.058)	1.009 (0.930, 1.090)	1.014 (0.937, 1.093)	1.053 (0.980, 1.129)
- Township	1.001 (0.925, 1.081)	0.982 (0.905, 1.061)	1.058 (0.983, 1.135)	1.000 (0.929, 1.073)
- Country	1.113 (1.032, 1.198)**	1.054 (0.975, 1.137)	1.115 (1.037, 1.198)**	1.046 (0.973, 1.123)
**Marital status** (ref. No spouse)	0.986 (0.905, 1.075)	1.010 (0.926, 1.102)	1.120 (1.029, 1.221)**	0.919 (0.850, 0.995)*
**Living alone** (ref. No)	0.976 (0.898, 1.057)	1.065 (0.985, 1.149)	0.999 (0.921, 1.081)	0.960 (0.889, 1.034)
**Financial status** (ref. Very insufficient**)**				
-Insufficient	0.959 (0.831, 1.114)	0.932 (0.806, 1.084)	0.987 (0.857, 1.144)	1.031 (0.896, 1.193)
-Sufficient	0.905 (0.760, 1.089)	0.919 (0.772, 1.106)	0.956 (0.804, 1.147)	0.989 (0.834, 1.186)
- Very sufficient	0.921 (0.797, 1.068)	0.965 (0.836, 1.119)	0.955 (0.829, 1.105)	0.964 (0.838, 1.114)
**With SPMSQ Error** (ref. No)	0.956 (0.882, 1.037)	0.972 (0.896, 1.055)	0.902 (0.835, 0.975)**	0.962 (0.892, 1.037)
**ADL difficulty**	1.190 (1.115, 1.270)***	1.169 (1.094, 1.248)***	1.127 (1.054, 1.203)***	1.105 (1.033, 1.179)**
**Number of disease**	1.491 (1.384, 1.606)***	1.403 (1.301, 1.513)***	1.398 (1.299, 1.504)***	1.320 (1.229, 1.417)***
Observations	6,472	6,472	6,472	6,472
Log Likelihood	-2,306.488	-2,280.636	-2,425.587	-2,573.941
AIC	4,652.976	4,601.272	4,891.173	5,187.882

Note: OR = Odds Ratio; CI = Confidence Interval; SPMSQ = Short Portable Mental Status Questionnaire; ADL = Activities of daily living; ER = Emergency Room. AIC = Akaike Information Criterion. ***p <.001. ** *p*<.01. * *p*<.05

From Tables 2, 3, 4 and 5, among the covariates, the number of diseases had a significant positive influence on all four healthcare utilization outcomes, and this effect was consistent across all years. The next most significant factor was ADL difficulty, which showed a significant positive correlation with the 2015 number of outpatient visits for general reasons, 2015–2017 ER visits, and 2015–2018 hospital admissions.

## Discussion

This study found that higher levels of emotional loneliness in 2015 were cross-sectionally and longitudinally associated with an increased likelihood of seeking outpatient services for mental health issues. Increased levels of emotional loneliness in 2015 were associated with more frequent outpatient visits for general reasons in the same year. Conversely, higher levels of social loneliness in 2015 were cross-sectionally associated with a reduced likelihood of seeking ER care.

According to our current knowledge, this study provides the initial evidence supporting the link between emotional loneliness and seeking healthcare services for mental health conditions. Loneliness is not only a public health issue but also creates mental health challenges. Past studies have already demonstrated the connection between loneliness and depression or dementia [29]. Moreover, loneliness leads to heightened neuroendocrine markers of stress and impaired neurodegeneration in the hippocampus and other brain regions crucial for emotional regulation and cognition [30]. As a chronic stressor, loneliness not only impairs the neuroendocrine and immune systems but also exhibits a strong correlation with suicide ideation, poor sleep quality and personality disorders [31]. The combination of these factors can increases the probability of older individuals accessing mental healthcare services. This study found that the presence of higher levels of emotional emptiness in 2015 significantly increased the likelihood of utilizing mental healthcare services in each year from 2015 to 2018. This suggests that emotional loneliness influences the utilization of outpatient care for mental health illnesses both in the short-term and long-term. As a result, this research contributes to the existing literature by further confirming that the emotional aspects of loneliness, such as feelings of emptiness, lack of affection, and the presence of negative emotions, play a more critical and enduring role in increasing the likelihood of utilizing mental healthcare services, compared to the absence of essential social relationships.

In line with previous research conducted in the United States, Canada, and China, this study found that loneliness was positively correlated with the number of outpatient visits [7]– [8, 10]. Previous research has suggested a possible explanation that is seeking physician services is a way for lonely older adults to gain social support or social interaction, in addition to medical treatment [10]. Outpatient appointments can be conveniently scheduled and covered by NHI without requiring a prior diagnosis or referral, making them an affordable and accessible option for lonely older individuals to engage in conversations with others [8, 10]. Nevertheless, this study provides further clarification that it is the emotional dimension of loneliness, rather than social loneliness that exhibits a cross-sectional positive correlation with the number of outpatient visits during a two-year period. In other words, lonely older adults seeking outpatient services may not do so due to the absence of social interactions and relationships. Instead, they may seek these services to obtain emotional comfort through being attended to, cared for, and listened to. This helps compensate for their lack of emotional closeness and longing for deep emotional connections and intimacy.

In contrast to prior studies, this study found that social loneliness reduces the utilization of ER services, whereas previous research has either shown that higher loneliness levels [5, 17] or a lack of social support result in a slight increase in ER utilization [32–34]. One possible explanation for the inconsistent findings in this research and previous studies is that earlier research treated loneliness as a single composite measure [5, 17], without separately examining the differences in the relationship between social and emotional loneliness and ER visits. Another possibility is the lack of available companions, which may result in them being less likely to be discovered in emergency situations and less able to access immediate assistance [32]. This makes socially isolated individuals more vulnerable and at a higher risk of adverse health outcomes due to limited access to ER services. Regarding the differences in results between this study and earlier research examining the relationship between social support or social contact and ER visits [32]– [33], these discrepancies may be attributed to the fact that social support is not synonymous with social loneliness. Previous research often measures social support based on the quantity or frequency of social contact. However, Hsu (2020) found that 8.1% of older adults in Taiwan reported loneliness while co-residing with others, and 6.9% experienced both loneliness and isolation despite living with others. These groups exhibited lower IADL levels and family satisfaction compared to those who were neither lonely nor isolated. This suggests that older adults may experience heightened loneliness due to unmet expectations from family. Given the high prevalence of marriage and co-residence alongside elevated loneliness rates, substantial social contact may not necessarily protect against feelings of social isolation or loneliness [35]. As noted by Hsu (2020), co-residing older adults who felt lonely or isolated also exhibited lower IADL levels [35]. Combining Hsu’s and this study’s findings, co-residing older adults who are socially lonely and have limited access to ER services may be particularly vulnerable compared to their non-lonely and non-isolated counterparts.

This study found that both emotional and social loneliness, in cross-sectional and longitudinal analyses, were not associated with the odds of hospital admission. This result aligns with Newall et al. (2015) but differs from Hallgren et al. (2016), Gerst-Emerson & Jayawardana (2015) using Western samples, and Jiang et al. (2018) and Zhang et al. (2018) using mainland Chinese samples [7–10, 16]. Two possible reasons may explain this inconsistency. First, based on Table 5, ADL difficulty and the number of diseases were the most consistent and dominant predictors of the likelihood of hospitalization across all years. Although previous studies identified significant associations between feeling lonely and hospitalization [8–10, 16], their p-values were very close to 0.05, indicating a marginal relationship. In our case, the relationship between emotional and social loneliness and hospitalization may have been cancelled out by the stronger influence of more critical predictors, such as ADL difficulty and the number of diseases. Another possible explanation is that, unlike outpatient or ER visits, which patients can access on their own, hospital admissions require a physician’s assessment, diagnosis, and approval. Therefore, loneliness, as an emotional state, may not significantly influence this form of healthcare, which is contingent upon the rational decision-making of a third party.

This study combines a national sample with longitudinal registry-based NHI data and categorizes loneliness into emotional and social dimensions, allowing us to investigate the long-term and short-term relationships between these two forms of loneliness and healthcare utilization among older adults in Taiwan. Additionally, this study explores the relationship between loneliness and various healthcare utilization patterns, emphasizing that different types of loneliness have distinct impacts on different healthcare services. Therefore, the influence of emotional and social loneliness on each type of healthcare service should be examined and discussed separately. Nevertheless, this study has the following limitations that should be addressed. Firstly, while NHI data provides relatively objective information compared to self-reporting, it can only reflect the actual usage of healthcare services by the respondents. It does not necessarily represent their genuine healthcare needs. In other words, NHI data also signifies the accessibility of healthcare services to the respondents. Individuals with legitimate healthcare requirements may not be included in the NHI data due to various other reasons that prevent them from seeking medical care. Secondly, this representative sample required the consent of respondents to link with NHI data. Among the 8,300 respondents in this 2015 TLSA sample, 84% granted permission for the linkage, comprising the actual analytical sample for this study. Nevertheless, attrition within this sample may impose limitations on the applicability of the analysis results to the broader older population in Taiwan. As those excluded from the analyses had higher social and emotional loneliness, poorer health, and greater healthcare utilization in 2015, the findings of this study are generalizable primarily to older adults with relatively favorable psychosocial and physical conditions and lower healthcare utilization patterns. Thirdly, the Andersen Behavioral Model [25] proposes a comprehensive list of predisposing, enabling, and need factors that may influence individual healthcare service utilization. However, due to limitations in the data structure, some factors were not included in the analysis of this study, such as health belief, BMI and so forth. Fourth, loneliness data was only available in the first year (2015) and was not repeatedly measured in the following four years. Thus, dynamic changes in loneliness, the presence of chronic loneliness, and their effects on healthcare utilization cannot be observed.

## Conclusion

This study found that emotional loneliness in 2015 had a significant cross-sectional and longitudinal impact, increasing the likelihood of outpatient visits for mental health reasons and cross-sectionally correlating with the frequency of outpatient visits for general reasons. Conversely, social loneliness in 2015 may reduce the likelihood of ER visits in both the short term. Loneliness should be recognized as a critical need factor in healthcare utilization, as both emotional loneliness and social loneliness have unique influences on different types of healthcare services. It is essential to simultaneously examine these two loneliness aspects to comprehensively understand the impact of loneliness on healthcare utilization. The results of this study highlight the importance of addressing loneliness to enhance the physical and mental well-being of older adults and ensure the effective utilization of limited healthcare resources. Therefore, professionals serving older adults should regularly assess their level of loneliness as a screening tool for their healthcare needs and utilization patterns. Additionally, in Taiwan, psychological counseling services for older adults are limited and not covered by the NHI. Therefore, for individuals experiencing high levels of emotional loneliness, it is essential to provide accessible psychological support services and to develop outpatient case management mechanisms. These measures could help prevent the overuse of general or mental health outpatient services as substitutes for psychological support, thereby reducing overall outpatient utilization. Finally, those with elevated levels of social loneliness should benefit from measures that provide companionship during ER visits, ensuring their equal access to ER services.

## Data Availability

No datasets were generated or analysed during the current study.

## Abbreviations

ER: Emergency Room
UCLA: University of California, Los Angeles
NHI: National Health Insurance
TLSA: Taiwan Longitudinal Survey on Aging
ICD-10: International Classification of Diseases, 10th Revision
ADL: Activities of Daily Living
OR: Odds Ratio
